# Isometric training at longer muscle–tendon complex lengths: A potential countermeasure to impaired neuro‐muscle–tendon function during space travel

**DOI:** 10.1113/EP092225

**Published:** 2025-08-10

**Authors:** Gerard McMahon, Andy Sanderson, Hans Degens

**Affiliations:** ^1^ Sport and Exercise Sciences Research Institute, School of Sport Ulster University Belfast UK; ^2^ Department of Sport and Exercise Sciences, Institute of Sport Manchester Metropolitan University Manchester UK; ^3^ Department of Life Sciences Manchester Metropolitan University Manchester UK; ^4^ Institute of Sport Science and Innovations Lithuanian Sports University Kaunas Lithuania

**Keywords:** adaptation, biomechanics, exercise, physiology, space

## Abstract

Manned space exploration to distant destinations, including Mars, continues to be an aspiration of humankind. Space travel does, however, present many challenges to the body, amongst which adaptation to microgravity is perhaps the largest. For instance, both short and long manned spaceflight missions have shown substantial deleterious effects on muscle size and neuromuscular function. Although the neuro‐muscle–tendon system is responding primarily to the load to which it is subjected, resistive exercise countermeasures with dynamic contractions during space travel do not entirely mitigate the space travel‐induced deteriorations in neuro‐muscle–tendon function, probably owing to a lack of overall accumulation of sufficient mechanical stress. The aim of this review is to evaluate the evidence for isometric resistance training at longer muscle–tendon complex lengths to mitigate microgravity‐induced deterioration in neuro‐muscle–tendon function better than conventional resistance‐training programmes. It has been shown that specific joint positions, associated with a longer muscle–tendon complex, require larger internal muscle forces for the same external torque, thus requiring more muscle activation and imposing more tendon strain than during conventional dynamic resistance training. Isometric resistance training also confers the advantage of requirement of less voluminous equipment, in comparison to that required for dynamic resistive exercise. This factor is particularly important for space travel owing to the physical space and mass constraints. In addition, isometric contractions allow for easier monitoring and progression in exercise prescription compared with dynamic contractions.

## Abstract (highlights)

- **What is the topic of this review?**
  Exposure to microgravity induces reductions in neuro‐muscle–tendon complex mass, properties and function. Current countermeasures, in terms of the nature and prescription of resistance exercise, have not mitigated losses completely. Specifically, researchers have identified the necessity to incorporate more higher‐intensity mechanical loading in microgravity.
- **What advances does it highlight?**
  Performing high‐intensity isometric resistance training at longer muscle–tendon complex lengths might potentially modulate losses of the neuro‐muscle–tendon systems in microgravity through optimization of muscle mechanical stress, muscle activation and tendon strain. This method also provides logistical benefits, including minimal equipment, training monitoring and exercise prescription.

## 1 INTRODUCTION

Worldwide government spending on space exploration reached $117 billion in 2023, and Deloitte estimates that private companies have invested another $282 billion in the space sector between 2013 and 2022 (https://www.deloitte.com/us/en/insights/industry/government‐public‐sector‐services/government‐trends/2025/space‐industry‐growth.html). Investment banking company Morgan Stanley has projected that the space industry, including space travel, will be a potential $1 trillion dollar industry by 2040 (https://www.morganstanley.com/Themes/global‐space‐economy), with aims to return humans to the moon and explore further to Mars. However, one of the main limiting factors to human space exploration is the negative impact of the space environment on health, including musculoskeletal health (Fitts et al., 2000). Microgravity (MG) induces a plethora of changes, such as a rapid atrophy/loss of muscle mass and loss of neuro‐muscle–tendon (NMT) function in astronauts (Fitts et al., 2010; Koryak, 2017; Trappe et al., 2009). A recent systematic review and meta‐analysis highlighted that during spaceflight lasting from only a few days to ∼6 months, humans experience large decreases in lower‐limb muscle mass and strength, irrespective of the countermeasures used in‐flight (Comfort et al., 2021). It is estimated that during a return spaceflight to Mars (including a 12‐month stay on Mars), humans might lose ≤50% of knee‐extensor strength, even with current spaceflight resistive exercise countermeasures (Carpenter et al., 2010). Therefore, effective, practical solutions to maintain muscle mass in space are crucial in allowing humans to remain in MG for an extended exploration period.

Under standard earth gravity (1G), resistance training is an effective intervention to increase muscle mass and NMT function because it increases net muscle protein synthesis (Damas et al., 2018) and motor unit activation (Heckman & Enoka, 2012) and enhances tendon mechanical properties (McMahon, 2022). During several spaceflight missions, it has been used concurrently with aerobic exercise, in the form of treadmill running and cycling, to mitigate NMT deterioration. However, current training practices aboard spacecraft include the use of the advanced resistive exercise device (ARED). The ARED is made up of a combination of pistons contained within vacuum tubes and flywheel cables to mimic more traditional Earth‐based inertial resistance exercise practices in the absence of gravity. Despite double the resistive force of its predecessor, the ARED has been less effective in mitigating the MG‐induced muscle atrophy and weakness in comparison to the previous interim advanced resistive device (iRED), which also did not entirely mitigate the MG‐induced muscle wasting and weakness. Comfort et al. (2021) highlighted numerous potential causes for the apparent lack of effectiveness of such devices, with amongst them perhaps the most important factor that the astronauts performed 6–15 repetitions at a load little more than their body mass. The authors point out that such a load is unlikely to be sufficient to maintain NMT mass and function and suggest that, instead, perhaps the exercise needs to be continued until the astronaut is unable to complete the repetition at the set load (Comfort et al., 2021; McMahon, 2024; Schoenfeld, Grgic et al., 2017). Although this strategy might be adequate to mitigate the MG‐induced loss of muscle mass and strength, it has been shown that it does not induce adaptations of tendon morphology, perhaps owing to a lack of sufficient tendon strain (Lambrianides et al., 2024). Even if space travellers were to adopt training to task failure to mitigate NMT system deterioration, the increased effort required might present problems with adherence to the training protocol, possible increased nutrient intake requirements and delayed neuromuscular recovery (Haun et al., 2017).

On Earth, resistance‐training methods often rely on gravity to impose a load on the muscle–tendon unit that will stimulate muscle growth that requires bulky, specialized equipment (e.g., dumbbells, barbells, machines and pulleys). The operating space within space‐bound vehicles is severely restricted and costly. Therefore, the size and mass of any equipment to perform resistance training in space need to be kept to a minimum while still being effective. Non‐gravity‐based isoinertial (flywheel) devices have been adopted in spaceflight to counter some of the issues of traditional resistance‐training set‐ups and were integrated into the ARED. As already mentioned, results from the ARED use have had limited and variable success in eradicating muscle mass and functional losses following MG exposure, even when astronauts typically spend 2.5 h each day on exercise interventions (Bosutti et al., 2025).

The aim of this review is to outline: (1) the NMT challenges faced by astronauts and future space explorers; and (2) potential resistance‐training techniques that might facilitate maintenance or even improve NMT function and mass during spaceflight.

## 2 NEURO‐MUSCULAR–TENDON IMPAIRMENTS FOLLOWING EXPOSURE TO MICROGRAVITY

### 2.1 Muscle size, morphology and architecture

The rapid effects of MG on muscle mass and function are startling. LeBlanc et al. (1995) showed that after only 8 days aboard the STS‐47 shuttle, astronauts experienced significant MRI‐assessed muscle volume losses of −6.3% in the soleus–gastrocnemius, −3.9% in anterior lower leg muscles, −8.3% in the hamstrings, −6% in the quadriceps and −10.3% in the intrinsic back muscles. After 2 weeks post‐flight recovery, hamstrings and intrinsic back muscle size were still below preflight levels. Additionally, Edgerton et al. (1995) showed that after only 5 days of spaceflight, the cross‐sectional areas (CSAs) of type I and type II fibres in muscle biopsies of the vastus lateralis muscle were 11% and 24% smaller than preflight, respectively. They found that after 11 days of spaceflight, this had progressed to 16%–36% atrophy, with the effect being type IIB > type IIA > type I, and there were 6%–8% fewer type I fibres than preflight. Widrick et al. (1999) found in muscle biopsies in four male astronauts from the soleus muscle that type I muscle fibres showed a 15% reduction in fibre CSA, accompanied by a 21% loss in absolute peak force 17 days post‐spaceflight compared with preflight. Interestingly, all but one of the astronauts showed a reduction in specific tension (force per CSA) that might be related to a lower filament density, which in all astronauts might have contributed to the ∼30% increase in unloaded maximal shortening velocity of type I fibres postflight. It was postulated that (given that power is determined by force and velocity) the 30% increase in maximal shortening velocity might have helped to offset some of the reductions in absolute peak power, which will have contributed to the maintained (or even increased, in one astronaut) power per CSA. Other observations included maintained Ca^2+^ sensitivity of single muscle fibres in three of the astronauts and a somewhat reduced sensitivity in one of the four astronauts. Taken together, these studies provide insight into the rapidly developing and substantial deleterious effects of MG on muscle size and function.

Studies with a longer duration of MG exposure provide an insight into the challenges of future astronauts and commercial space travellers undertaking more extensive journeys through space. Most studies have focused on the NMT function of the triceps surae muscle group, owing to its role in weight‐bearing and the substantial effects of MG highlighted above. MG exposures of 90–380 days have revealed large reductions in muscle volumes of individual muscles of the triceps surae group. MG might impact the soleus muscle to a greater extent than the gastrocnemius, with soleus muscle volume losses of −15% to −19%, in comparison to −10% to −13% in the gastrocnemius muscle (Gopalakrishnan et al., 2010; Rittweger et al., 2018; Trappe et al., 2009). Other indices of triceps surae muscle morphology changes include a 20% atrophy of soleus type I muscle fibres (Fitts et al., 2010), reductions in gastrocnemius physiological CSA of between −6% and −11% (Rittweger et al., 2018), and reductions in muscle thickness of the medial gastrocnemius (−42%), lateral gastrocnemius (−47%) and soleus (−46%) (Koryak, 2019). It is noticeable, however, that these declines are similar in magnitude to the 6% atrophy of the triceps surae after 8 days of spaceflight (LeBlanc et al., 1995), the 15% fibre atrophy of the soleus muscle after 17 days of spaceflight (Widrick et al., 1999) and the 16%–36% reduction of fibre CSA after 17 days of spaceflight (Edgerton et al., 1995). This thus suggests that the muscle (fibre) atrophy occurs rapidly, during the first days of spaceflight or bed rest (Hendrickse et al., 2022), and might then reach a so‐called ‘default muscle mass’, as seen after denervation in animal models (Degens et al., 2008; Paudyal et al., 2018; van der Meer et al., 2011).

Muscle architecture, the spatial arrangement of muscle fibres relative to their force‐generating axis (Lieber & Friden, 2000), is also impacted by MG. The changes in architecture include 33%–53% reductions in fascicle length and pennation angle across various ankle joint angles following 180 days on the International Space Station (Koryak, 2019).

### 2.2 Neuromuscular impairments

A further aspect crucial to muscle function is the neural input to the muscle. Lambertz et al. (2001) reported that the maximal motor direct response (*M*_max_) normalized root mean square values of EMGs decreased by −22% to −68% and EMG amplitude by −39% during a maximal voluntary contraction (MVC) of the triceps surae. However, in this study, the preflight MVC was used to normalize the postflight data and, given the substantial changes in muscle architecture during spaceflight and the time passed (110–200 days), this method of normalization is no longer considered appropriate compared with more modern standards (Besomi et al., 2020). Koryak (2017) showed that soleus integrated EMG and the EMG/MVC ratio increased by 55% and 71%, respectively, indicating a larger neural input for a given force output following extended MG exposure. Such an increase in EMG/force might be a consequence of the increase in surface‐to‐volume ratio during atrophy, hence a larger action potential per unit of fibre volume. That such a situation is potentially significant is reflected by the larger energy expenditure for membrane repolarization (Na^+^/K^+^‐ATPase pump) in juveniles than in the adult lobster, *Homarus americanus* (Jimenez et al., 2011). It is, however, somewhat puzzling that Koryak (2017) also reported a significant decline in the slope of the EMG–force relationship postflight, which must mean that during submaximal contractions there is less neural input (rather than more neural input, as during the MVC) for a given force output. Another indication that neural function in humans following spaceflight is somewhat reduced comes from the observation that the loss in muscle function assessed via MVIC far exceeds that of muscle volume loss (Fitts et al., 2000). Besides a decreased neural drive to the muscle or changes in motor unit recruitment patterns (Lambertz et al., 2001), this might also be attributed to a reduction in specific tension, as seen in single fibres after space flight of short duration (17 days; Widrick et al., 1999), but not long duration (180 days; Fitts et al., 2010). In summary, disentangling potential neural impairments from changes at the muscle level is difficult based on the available, limited datasets in humans.

### 2.3 Tendons

We are not aware of studies conducted in humans following spaceflight that have directly measured tendon–aponeurosis or free tendon mechanical properties using established, appropriate methods (Seynnes et al., 2015). The only study that has attempted to estimate muscle–tendon complex stiffness following spaceflight was the one by Lambertz et al. (2001). The authors estimated changes in muscle–tendon complex stiffness via a series of quick‐release tests and calculated stiffness as the ratio between variation in angular acceleration and angular displacement multiplied by the corresponding inertia value in the first 20 ms of muscle contraction. The slope of the linear stiffness–torque relationship was then used as an index of musculotendinous stiffness. Using this technique, the authors reported a 25% increase in the musculotendinous stiffness index following spaceflight. However, there are several potential issues with this observation. The methods used were adapted from classical methods used to calculate stiffness in series elastic components in isolated muscle instead of an in vivo muscle–tendon approach using B‐mode ultrasonography, EMG and force recordings. As such, the reliability of this calculation is unknown, and it is difficult to ascertain whether the values are accurate.

The electromechanical delay is the time interval between the onset of muscle activity (EMG) and the onset of muscle force detection and encompasses the time from arrival of the action potential at the neuromuscular junction to the moment of force development. It encompasses not only the time required for excitation–contraction coupling, including action potential propagation, the release of calcium from the sarcoplasmic reticulum and cross‐bridge cycling, but also force transmission through serial sarcomeres, intramuscular connective tissue and tendon. In Earth‐based populations, it is known that stretching of in‐series elastic components (i.e., the tendon–aponeurosis and tendon) accounts for most of the variation in the electromechanical delay (Nordez et al., 2009). Indeed Koryak (2017, 2019) concluded that the 34% increase seen in electromechanical delay accompanied by a 36% reduction in rate of force development was down to a decrease, rather than an increase (Lambertz et al., 2001), in muscle–tendon stiffness. Reeves et al. (2005) also showed that after 90 days of bed rest, gastrocnemius tendon stiffness decreased, rather than increased, by 58%, modulus by 57% and tendon stress by 27%, whereas maximal strain increased by 27% despite ∼30% decrease in maximal force. These authors also showed that although isoinertial flywheel training of the triceps surae muscle group attenuated reductions in tendon stiffness (37% vs. 58%), modulus (38% vs. 57%), tendon stress (14% vs. 27%) and maximal strain (17% vs. 27%), they were still not entirely ameliorated. This is in agreement with other studies showing substantial reduction in tendon stiffness following periods of detraining (Kubo et al., 2010; McMahon et al., 2014).

The above sections highlight that both shorter and longer exposures to MG result in large reductions in muscle size and NMT function, although all modern missions include resistive exercises as countermeasures. This presents a serious challenge to space agencies and commercial entities with goals of furthering space exploration and highlights a need for novel mitigation approaches. Researchers have called for the deployment of higher‐intensity muscle contractions and altered training prescription to mitigate MG‐induced losses more effectively in NMT structure and function (Comfort et al., 2021; Fitts et al., 2010; Trappe et al., 2009).

### 2.4 Muscle function

Accompanying the decrements in muscle size, the maximal force of voluntary isometric contraction (MVIC) of anti‐gravity and postural muscles, such as the triceps surae, fell between −17% and −42% (Gopalakrishnan et al., 2010; Koryak, 2017, 2019; Lambertz et al., 2001; Rittweger et al., 2018), knee extensors −15%, knee flexors −20%, hip extensors −15% and hip flexors −28%, while the loss of muscle strength was much less in non‐antigravity muscles, such as the elbow extensors (−11%) and elbow flexors (−8%) in spaceflights lasting 90–180+ days (Gopalakrishnan et al., 2010). Interestingly, in one study, it was reported that when the MVIC was reduced by 42%, the maximal tetanic force electrically evoked via tibial nerve stimulation had decreased by only 26% in seven cosmonauts following 213 ± 30 days of spaceflight (Koryak, 2017). This suggests that there is, in addition to muscle atrophy, also an impaired ability to recruit the remaining muscle tissue voluntarily.

In addition to isometric function, dynamic muscle performance is potentially even more severely impacted by extended MG exposure. Although the similar reduction in isokinetic strength in the knee flexors (−24%), knee extensors (−10%), ankle plantarflexors (−22%) and ankle dorsiflexors (−8%) in comparison to MVIC discussed above suggests that this might not be the case (Gopalakrishnan et al., 2010), the 32% loss in peak power in comparison to a 20% reduction in maximal isometric force (Trappe et al., 2009) does suggest that dynamic performance is affected more than isometric performance. This might be attributable to a 36% reduction in the rate of force development (normalized to MVIC) (Koryak, 2017) and a 34% increase in electromechanical delay (Koryak, 2017, 2019). These global changes in function at the whole‐joint level are underpinned by changes at the cellular level. In contrast to the increased maximal shortening velocity after 17 days of spaceflight that was related to a decreased thin filament density (Widrick et al., 1999), it was reduced after 180 days of spaceflight and was accompanied by an increased thin filament density (Fitts et al., 2010). There thus seems to be a transient increase in maximal shortening velocity, followed by a decrease in shortening velocity that could contribute to the MG‐induced decline in muscle power. However, the slow‐to‐fast fibre‐type transitions in myosin heavy chain (MHC) phenotype attenuate the decline in power, because fast type II fibres can produce about six times as much power as similar‐sized type I fibres (Gilliver et al., 2009). For instance, Trappe et al. (2009) showed that after 6 months aboard the International Space Station, there was a 12% decrease in gastrocnemius in MHC type I fibres and an increase in MHC I/IIa hybrid fibres (4%) and MHC IIa fibres (9%), with minimal MHC IIx fibres detected pre‐ or postflight, whereas in the soleus there was a 17% decrease in MHC type I fibres, with no significant change in hybrid phenotypes (MHC I/IIa, IIa and IIa/IIx). Together, these findings would suggest a potential increase in power output per unit muscle mass following spaceflight, which is probably negated by the often observed decrease in specific tension.

A possible explanation for the increased electromechanical delay, in addition to the role of changes in the in‐series elastic component compliance (discussed in Section 2.3), is that excitation–contraction coupling might be negatively impacted. Although in one astronaut in the study by Widrick et al. (1999), the Ca^2+^ sensitivity of single muscle fibres was slightly reduced, there was no significant change in the other three astronauts.

## 3 EVOLUTION OF RESISTANCE‐TRAINING PRACTICES IN MICROGRAVITY

Despite substantial progress, current and historical resistance‐training prescriptions (outlined briefly in Table 1; for a more detailed review, see Hackney et al., 2015) show several limitations that are likely to limit their ability to preserve muscle mass and NMT function fully in space. Many of the earlier resistance‐training programmes using the iRED device included training with moderate loads, because the total resistive load of the device was limited to 136 kg. Therefore, for an astronaut weighing 90 kg, only a further 46 kg of external load could be added to the system above the astronaut's body mass. In fact, the astronauts self‐reported that the loads were ‘too light’ (Trappe et al., 2009). To overcome this limitation, single‐legged squat variations were added to the prescribed lower‐body resistance exercises, in addition to bilateral squat and deadlift variations and heel lifts (Loehr et al., 2015). Besides the limited load, astronauts also completed only16–20 repetitions per set at an intensity of ∼60%–65% 1RM, a training load that did not lead to muscular failure, even momentarily.

**TABLE 1:** Brief modern history of resistance training in space.

Era	Device	Key features	Typical prescription	Limitations
Shuttle Era International Space Station pre‐2008	Interim resistive exercise device (iRED) iRED + treadmill and cycle ergometer	Elastic cable‐based loading; 29 resistance exercises Multimodal training, low mechanical specificity	6–20 repetitions, 3 sets, varied load	Low loading capacity (<136 kg); inconsistent resistance Minimal high‐load strength training
International Space Station post‐2008	Advanced resistive exercise device (ARED)	Simulates free‐weight barbell loads via vacuum cylinders Flywheel cables	6–15 repetitions, 3–5 sets, load based on preflight repetition maximums (i.e., 6, 8, 12 +75% body weight), consisting of light, moderate and heavy days	Bulky; limited by bar path and dynamic loading variability

Further limitations of earlier devices were concerns about the risk of injury and hardware fatigue/failure. For example, on one mission, the iRED was unavailable to astronauts for 1 month owing to equipment failure. Later missions sought to overcome these primary limitations by increasing the maximal load capacity from 136 to 272 kg in the ARED. In addition, dynamic exercises (similar to previous exercises used in the iRED) are now also prescribed that target lower/upper limbs, hip trunk and upper body musculature. Despite this increased load capacity and targeting more muscle groups, the ARED (athough effective in some) was still not effective in mitigating muscle mass and function loss in most astronauts, even with high protocol compliance (Trappe et al., 2009). This suggests high inter‐individual variability in the response to resistive countermeasures and the potential need for alternative strategies to be developed and/or reflects a genetic predisposition to be a responder or non‐responder to specific resistance‐training interventions (Erskine et al., 2010, 2014)

There are currently different resistance‐training programmes in space travel being trialled, such as jump exercises and flywheels with loading. However, these have not been trialled in an astronaut population during prolonged MG exposure, hence it is too early to comment on their efficacy. To date, high‐force isometric exercises of any kind have not been prescribed or used by astronauts despite their terrestrial success in developing muscle mass. Given this success on Earth, it is desirable to assess the efficacy of high‐force isometric exercise to mitigate MG‐induced reductions in NMT properties and function. Using biomechanics to identify high‐force‐generating isometric exercises, which commonly involve training at longer muscle–tendon complex lengths, might be an even more specific way of targeting the desired adaptations.

## 4 ISOMETRIC TRAINING AT LONGER MUSCLE–TENDON COMPLEX LENGTHS: RATIONALE FOR THE APPROACH

### 4.1 Muscle size, morphology and architecture

Muscle is a mechanosensitive tissue, with tension being the primary stimulus for muscle adaptation via activation of various mechanotransduction signalling pathways (Roberts et al., 2023; Wackerhage et al., 2018). Muscle contractions can be subdivided into static (isometric) or dynamic (concentric and eccentric) contractions. Isometric contractions involve a contraction where neither the external joint angle nor the internal length of the muscle–tendon complex as a whole changes. However, the individual constituents themselves (muscle and tendon) do undergo some change in length during an isometric contraction, whereby muscle fascicles will shorten and the tendon will elongate, as seen with ultrasonography of the muscle–tendon complex during submaximal and maximal MVIC (Fukunaga et al., 1997; McMahon et al., 2013). In terms of maximal muscle force, the contraction type magnitude follows the order eccentric > isometric > concentric. Given that the musculoskeletal system is a system of muscle–tendon actuators acting across joints as levers, muscles cause movement mainly by bringing about joint rotation (Sherman et al., 2013). As a joint rotates during the performance of a resistance‐training exercise, this creates both internal and external joint moments. These moments are calculated with the formula: Moment = *F* × *D*, where *F* is the force (in newtons) and *D* is the length of the moment arm (in metres), which produces a moment of torque measured in newton metres (N m). The moment arm is defined as the perpendicular distance from the line of force application to the (joint) centre of rotation (Sherman et al., 2013). Based on literature pertaining to internal joint moments in humans combined with an analysis of the force–length (or torque–angle) curves of a muscle group, we can develop exercises at specific joint angles that will maximize the mechanical stress conveyed to the muscle fibres.

For example, let us look at the potential effect of the mechanical stress on the knee extensors by adopting two different joint angles (50° and 90° of knee flexion, 0° = full extension). The torque–angle relationship of the knee extensors extends over the ascending, plateau and descending limb phases, with peak torque at ∼70° knee flexion and almost identical external torques produced at both 50° (ascending limb) and 90° (descending limb) of knee extension during an MVIC (McMahon, Best et al., 2022; McMahon & Onambele‐Pearson, 2024). However, owing to the length of the internal patella tendon moment arm at 90° knee flexion being <50% of the moment arm length at 50° knee flexion (Krevolin et al., 2004), internal muscle force production has been shown to be 35% higher at 90° than at 50° for the same external isometric torque (McMahon & Onambele‐Pearson, 2024). Past 90° knee flexion, the internal moment arm does not change much further; however, the knee extensor torque will decrease further along the descending torque–angle relationship (Marginson & Eston, 2001) and therefore internal forces will be lower. Although this is only an example of joint angles and muscle forces, it demonstrates that it is possible to individualize and optimize the mechanical tension to the muscle in MG based on a biomechanical approach. In comparative terms with dynamic training, dynamic training moves the muscle along its length–tension curve, requiring it to operate at both lower and higher force‐generating lengths and activation states. Also, according to the force–velocity relationship of muscle, dynamic contractions involve considerably less muscle force than isometric contractions. Periodically moving to joint angles that have lower force generation and muscle activation might not optimize the conditions for muscle growth. For example, Pedrosa et al. (2022) demonstrated that performing resistance training at longer muscle lengths exclusively was superior to a full range of motion for eliciting muscle hypertrophy in the quadriceps.

Alegre et al. (2014) found that after 8 weeks of isometric training of the knee extensors at either a shorter (50° knee angle) or longer (90° knee angle) muscle length, vastus lateralis muscle thickness was increased along the entire length of the muscle after training at the longer muscle length, whereas only proximal and central increases in thickness were seen after training at shorter muscle lengths, as suggested by the significant time × group interaction at the central muscle location. Furthermore, Noorikov et al. (2014, 2015) showed that after 6 weeks of isometric training at shorter (∼38° knee flexion) and longer (∼87° knee flexion) muscle lengths, the quadriceps volume and CSA of the individual quadriceps muscles improved significantly after training at longer but not shorter muscle length (muscle volume ∼6% vs. −0.5%). These findings are similar to those for dynamic contractions with a similar training duration, muscle group, muscle location and training angles (McMahon et al., 2014a).

Isometric resistance training provides comparable muscle adaptations to other contraction types (concentric and eccentric). In the only human study comparing the impact of 12 weeks of concentric, isometric and eccentric resistance training, it was found that the change in the quadriceps muscle CSA (4%–6%) did not differ between these training programmes (Jones & Rutherford, 1987). This is supported by animal molecular data showing that after 20 days of loading with isometric, concentric or eccentric contractions, muscle mass increased by 14%, 12% and 11%, respectively, with similar changes in muscle DNA and RNA content (Adams et al., 2004).

In addition to muscle size, architectural adaptations are superior to training isometrically at longer versus shorter muscle lengths, with increases in pennation angle found after training at longer muscle length, but none found after training at shorter muscle length, and no differences found in fascicle lengths between training conditions (Alegre et al., 2014). What is also important to note is that these muscle adaptations after isometric training at longer muscle lengths were achieved with relatively low‐volume and moderate‐ to high‐intensity contractions. For example, during the first 4 weeks of training, only six to nine sets composed of six or seven repetitions of 5 s isometric contractions at 60%–70% MVC were completed, followed by a further 4 weeks of training at 80% MVC for four sets of five or six repetitions, two or three times per week. Such relatively low‐intensity, low‐volume training programmes might be attractive to astronauts/cosmonauts and others owing to other mission pressures/priorities and motivation to train (Comfort et al., 2021) and, at the same time, be more effective than current resistive countermeasures to mitigate MG‐induced muscle atrophy and weakness. It is to be expected that a greater volume and higher intensity (close to failure) with longer rest intervals of training isometrically at longer muscle–tendon complex lengths might be even more effective (Grgic et al., 2021; McMahon et al., 2022b; Schoenfeld, Grgic et al., 2017).

### 4.2 Muscle function

Another well‐established benefit from performing isometric training at longer muscle–tendon complex lengths is that it increases muscle strength over a wider range of joint angles than training at shorter lengths. For example, Kubo et al. (2006) showed that muscle strength was significantly greater after 12 weeks of isometric training over eight knee angles from 40° to 110° knee flexion in the 100° training leg (longer length), whereas the contralateral leg that was trained at 50° (shorter length) experienced strength gains only at short‐to‐moderate muscle lengths (knee angles of 40°–80° knee flexion). Furthermore, Alegre et al. (2014) showed that dynamic (isokinetic) strength was significantly increased after training at longer muscle lengths (+23.5% ± 11.4%), but not after training at shorter muscle lengths (+10% ± 12.4%). They also found that the optimal torque‐angle shifted to longer and shorter muscle lengths after training at longer and shorter muscle length, respectively. This is probably attributable to addition or removal of sarcomeres in series during training at short and long muscle lengths, respectively, as seen during casting at short or long length in animal studies (Tabary et al., 1972). Another factor is the muscle‐length specificity of neural adaptations after isometric training, where muscle activity is increased only close to the training joint angle for shorter muscle lengths, whereas it is increased across a wider range of joint angles after training at longer muscle lengths (Kitai & Sale, 1989; Thepaut‐Mathieu et al., 1988). Also, in the aforementioned studies of Noorikov et al. (2014, 2015), after 6 weeks of isometric training at shorter and longer muscle lengths, MVIC and peak concentric torque at slower and faster angular velocities were greater after training at longer compared with shorter muscle lengths. These studies also showed relationships between the changes in muscle size induced at longer muscle length and changes in maximal isometric strength and torque at various angular velocities.

In addition to the muscle‐length‐specific nature of the muscular and neural adaptations of isometric training at different muscle lengths, muscle activity itself has been shown to be higher systematically during isometric resistance exercise at longer versus shorter muscle lengths. Several authors have shown that during resistance exercise, when external torques are matched and even when internal muscle forces are matched, training at longer muscle lengths produced significantly higher EMG amplitudes and voluntary activation than at shorter muscle lengths (Bampouras et al., 2006; Kluka et al., 2015; Kooistra et al., 2006, 2005; Marchetti et al., 2016; McMahon, 2024), indicating that the requirement of muscle fibres to produce force at longer muscle lengths requires greater motor unit recruitment or rate of action potential discharge. Also, maximal isometric contractions themselves have been shown to produce significantly greater muscle activation than both maximal eccentric and concentric contractions in the knee extensors (Babault et al., 2001). Therefore, muscle length and contraction type might be key mediators of neuromuscular adaptations that can be harnessed for improving motor unit recruitment during prolonged MG exposure.

### 4.3 Tendons

Tendons work in tandem with muscle, acting on the bone to bring about concerted joint rotation (Ker et al., 1988; McMahon & Cook, 2024). The synchronization of tendon and muscle adaptation is crucial to preserve NMT function, prevent injury and maintain tendon health (Arampatzis et al., 2020; McMahon et al., 2013; Mersmann et al., 2017). The mechanical and material properties (Bohm et al., 2015; Maganaris et al., 2004; McMahon et al., 2018) and their morphology (Kongsgaard et al., 2007; Seynnes et al., 2009) are changed in response to mechanical loading via enhanced expression of extracellular matrix components that is mediated by growth factors (Heinemeier & Kjaer, 2011; Heinemeier et al., 2013; Kjaer et al., 2009). These adaptations appear to be a graded response to mechanical loading, whereby mechanical loading (tendon strain) below a particular threshold does not induce mechanical or morphological changes (Arampatzis et al., 2007, 2010; Lambrianides et al., 2024; McMahon, 2022a). In terms of muscle–tendon complex length, several studies have shown that performing both dynamic (McMahon et al., 2014b) and isometric (Kubo et al., 2006; Lambrianides et al., 2024) training at longer muscle–tendon complex lengths is superior to shorter muscle–tendon complex lengths for increasing tendon mechanical and material properties, even when internal forces/moments are matched. Kubo et al., 2006 showed that after 12 weeks of isometric training at shorter (50° knee flexion) and longer (100° knee flexion) muscle–tendon complex lengths the patella tendon stiffness was significantly increased (∼52%) for the longer muscle–tendon complex length, with no changes at the shorter muscle muscle–tendon complex length. Likewise, Lambrianidis et al. (2024) found that Achilles tendon stiffness, modulus and CSA were all increased to a greater extent after 12 weeks of training at a longer muscle–tendon complex length (85° dorsiflexion) compared with a shorter muscle°tendon complex length (115° plantar flexion). Perhaps a larger strain, when training at longer lengths, is the underlying factor for the better results in comparison to training at short lengths. This supports current recommendations to perform high‐intensity contractions of 70%–80% MVC or greater to generate sufficient tendon strain (Arampatzis et al., 2020; McMahon, 2022) to maximize mechanical and morphological adaptation in tendons (Bohm et al., 2015; Lazarczuk et al., 2022). Isometric contractions appear to be superior to concentric contractions carried out at the same relative intensity for improving tendon mechanical properties (Kubo et al., 2009). This is likely to be because the total mechanical strain at common joint positions during isometric contractions is higher relative to dynamic contractions, where the tendon will move through regions of higher and lower mechanical strain throughout a full range of motion owing to the force–length properties of muscle, internal moment arms changes and passive strain on the muscle–tendon complex (Herbert et al., 2002; Visser et al., 1990). These data indicate that high‐intensity contractions at longer muscle–tendon complex lengths could be an effective method for maintaining or improving tendon function, performance and health in MG.

### 4.4 Practical advantages and exercise prescription

One of the largest benefits of isometric training, with specific reference to space travel, is that it is simple and does not require complex and voluminous equipment. This is important because not only is space within a spacecraft precious and costly, but there is also a significant cost related to the transport of equipment. In addition, equipment must be safe, effective, stored efficiently and easily maintained. The current ARED system is large and complex, including the use of vacuum cylinders and isoinertial flywheels. Other recent suggestions as countermeasures for muscle mass loss include a horizontal sledge rack with moving carriages and tensile springs for jumping (Cleather & Kennett, 2024). However, jumping is not as effective as high‐force isometric exercise for maximizing motor unit recruitment or developing tendon mechanical properties (Enoka & Duchateau, 2017; Lazarczuk et al., 2022).

Isometric resistance exercise aboard a spacecraft would require only a steel frame (rack) with adjustable hooks, a bar and the floor of the spacecraft or a small platform, because the person acts in positions of constraints between themselves, the floor and the bar/frame. All major muscle groups can be targeted effectively by any force from 1% to 100% MVC with many different types of exercise. Adjustable hooks mean that the astronauts are not limited to exercise at long muscle–tendon complex lengths but can change to other joint angles that might be specific to other functions or to reduce strain. Furthermore, one could also prescribe exercises that are force based or ballistic in nature. Performing force‐based or ballistic isometric exercises results in distinct neuromuscular adaptations and functional changes (Tillin & Folland, 2014), such as a much more pronounced increase in the rate of motor unit recruitment, number of action potential discharges and rate of force development (Del Vecchio, 2023).

In terms of exercise prescription, isometric training affords huge versatility in exercise choice, exercise intensity (submaximal or maximal) and the nature of exercise (steady state, maximal force or ballistic). One might argue that high‐intensity isometric resistance training is less effective than dynamic contractions to elicit meaningful improvements in dynamic human movement. However, there is an ever‐growing body of empirical evidence demonstrating that isometric exercise does elicit significant improvements in dynamic performance tasks. Indeed, a recent review highlighted that chronic resistance training using solely isometric contractions did improve squat and counter‐movement jump performance, running, sprinting, sprint acceleration, sprint cycling, cycling, and both concentric and eccentric torque generation at different speeds (Lum & Barbosa, 2019; Lum et al., 2023, 2021). Perhaps as a result of this evidence, isometric resistance training is currently recommended for enhancing deceleration performance (Harper, 2023). The functional benefits of isometric training to dynamic performance are likely to be underpinned by the isometric training‐induced improvements in voluntary activation and tendon stiffness that will also result in a faster early rate of force development (RFD) (Kubo et al., 1999; Tillin & Folland, 2014). In addition, it should be noted that many dynamic actions are relatively isometric in nature in vivo, owing to the decoupling of joint actions and muscle–tendon behaviour (Lai et al., 2014). In many dynamic actions, in vivo muscle length changes are rather small or non‐existent as the tendon lengthens, resulting in many cases in isometric and quasi‐isometric contractions (Oranchuk et al., 2019; Tecchio et al., 2024). This then suggests that, contrary to intuition, isometric training is not inherently non‐specific, but rather, specificity can largely be engineered by manipulating joint angle, contraction intensity and intent. As such, it should be considered a viable and effective alternative modality to dynamic training.

Although the present review proposes the use of high‐intensity contractions at longer muscle–tendon complex lengths to maintain NMT characteristics and function, a broader use of isometrics across joint angles is warranted, which might help to avoid common MG‐induced injury risks (such as articular cartilage damage) that can also occur when training sessions are always performed at the same muscle–tendon length.

In a similar fashion to dynamic exercise, isometric exercise also allows the assessment of progression to be standardized. One could embed a force plate into the floor of the spacecraft where the resistance exercise takes place. This could be used to track MVICs or ballistic contractions of individual muscle groups or compound exercises (e.g., isometric squat, isometric mid‐thigh pull) in standardized conditions. The force plate can provide many mechanical variables during these contractions to provide valuable insight into gross NMT system function (Merrigan et al., 2021). There is also the opportunity for simple and effective biofeedback from force plates during isometric contractions, providing the individual with important information on training progression or attaining the desired target force levels during the exercise, thus enabling a feedback loop of exercise prescription → exercise monitoring → exercise testing → exercise prescription. Such isometric performance biofeedback of progression (or regression) could increase engagement, compliance and adherence to the training programme, which historically has been poor with astronauts/cosmonauts (Comfort et al., 2021).

Finally, training at joint angles that maximize mechanical stress during high‐force isometric contractions might also have implications for resistance‐training prescription. The most obvious target is training intensity, where more forceful contractions might be able to bring about the necessary adaptations with a lower volume, be it on the basis of a single exercise, per session or total weekly volume. Low‐load training (taken close to or to momentary muscle failure) requires significantly higher training volumes than higher‐load training over the course of a training programme (Schoenfeld et al., 2016), and although resulting in a similar increase in muscle size, gains in strength are inferior (Schoenfeld, Grgic et al., 2017). Furthermore, as long as sufficient tendon strain is achieved during an exercise, improvements in tendon mechanical properties are independent of training volume (Bohm et al., 2015; Lazarczuk et al., 2022). Again, this might help to improve recovery from resistance training, permitting higher frequencies of resistance training to be performed and aiding in adherence to the resistance‐training programme (Comfort et al., 2021; Haun et al., 2017).

Despite the potential benefits of isometric training at longer muscle–tendon complex lengths in MG based on the benefits seen in terrestrially based research studies, the efficacy of such training programmes has not yet been explored in the MG environment. The MG experienced in space, along with all other constraints of spacecraft, are not easy to replicate on Earth, but initial studies might be performed as bed‐rest studies.

## 5 POTENTIAL ADVERSE EFFECTS AND CONSIDERATIONS FOR ISOMETRIC RESISTANCE TRAINING IN MICROGRAVITY

One of the primary concerns of isometric exercise involves the cardiovascular system. Isometric contractions provoke significant increases in intrathoracic pressure and arterial blood pressure owing to the mechanical occlusion of vessels during muscle contractions. In the presence of fluid shifts in MG, the pressor response might be amplified, and during prolonged spaceflight and repeated exposures to isometric exercise, potentially diminish baroreceptor sensitivity and hence autonomic regulation of heart rate and vascular tone (Hughson et al., 2012). This, then, might aggravate the risk of syncope in astronauts and postflight orthostatic intolerance. In parallel, elevated intrathoracic pressure during high‐effort isometric manoeuvres can increase the already elevated intracranial and intraocular pressures even further, which is of particular concern in the context of spaceflight‐associated neuro‐ocular syndrome. Repeated exposure to elevated pressures might exacerbate symptoms such as optic disc oedema and visual impairment, conditions previously documented in long‐duration space missions (Mader et al., 2013). However, these challenges are present with high‐intensity dynamic contractions also, and it remains to be seen whether isometric exercise would necessarily exacerbate these conditions over and above that of dynamic contractions.

Furthermore, isometric loading might impose prolonged compressive forces on joints without the dynamic fluid exchange typically facilitated by movement. This mechanical environment might negatively affect articular cartilage, particularly in MG, where reduced synovial circulation and unloading already contribute to cartilage atrophy and degeneration (Fitzgerald, 2017). In this context, repetitive or sustained isometric contractions at extreme joint angles could accelerate the destruction of articular cartilage. The good news is that this can be largely, if not totally, mitigated by manipulating the training prescription to include short‐duration isometric contractions at multiple joint angles.

## 6 PERSPECTIVES

Current resistive exercises and exercise prescription have been unable to mitigate losses in neuromuscular and (likely) tendon function entirely, in addition to the slow‐to‐fast fibre‐type transition during brief and prolonged space exploration. Isometric training at longer muscle–tendon complex lengths might provide an effective and practical mechanically based solution as a countermeasure to MG‐induced losses in NMT function and mass in humans. Isometric muscle training at long muscle length could allow space travellers to target specific aspects of tissue adaptation with an overall lower training volume than current practices. Other beneficial aspects of this type of isometric training in comparison to current methods are the potential for training monitoring, testing, prescription and progressive overload. However, this approach needs to be examined further in terrestrial environments mimicking the space environment (such as a bed‐rest study) and explored further in the MG environment to gauge its full efficacy as a countermeasure to mitigate MG‐induced muscle mass and NMT functional decrements.

## AUTHOR CONTRIBUTIONS

Gerard McMahon conceived the review and prepared the original draft. Gerard McMahon, Andy Sanderson and Hans Degens reviewed and edited the manuscript. All authors approved the final manuscript and agree to be accountable for all aspects of the work in ensuring that questions related to the accuracy or integrity of any part of the work are appropriately investigated and resolved. All persons designated as authors qualify for authorship, and all those who qualify for authorship are listed.

## CONFLICT OF INTEREST

None declared.
